# Remote wet lab training in corneal surgery at LV Prasad Eye Institute in India

**Published:** 2023-12-01

**Authors:** Kavya Chandran, Karthikesh Anche, Pravin Vaddavalli, Padmaja Kumari Rani

**Affiliations:** 1 Shantilal Shanghvi Cornea Institute: LV Prasad Eye Institute, Hyderabad, India.; 2 Centre for Technology and Innovation: LV Prasad Eye Institute, Hyderabad, India.; 3 Shantilal Shanghvi Cornea Institute: LV Prasad Eye Institute, Hyderabad, India.; 4 Anant Bajaj Retina Institute: LV Prasad Eye Institute, Hyderabad, India.

**A low-cost remote wet lab model developed during the COVID-19 pandemic continues to be useful, eliminating the need for surgical fellows to travel long distances.**

Tele-mentoring and remote surgical training can effectively bridge geographical distances, provide expert-led education, and foster equitable learning opportunities.^1,4^

LV Prasad Eye Institute (LVPEI) is a multi-tier eye care network across southern and eastern India. It includes a centre of excellence, three tertiary centres, and 26 secondary centres. The centre of excellence delivers advanced subspeciality care to 50 million people, and tertiary centres provide this care to 5 million people. In the rural areas, the secondary centres focus on providing comprehensive ophthalmology and cataract surgical care.

Education, encompassing resident and fellowship training, is an important cornerstone of LVPEI. The Cornea Fellowship Programme initially spanned 24 months: 22 months of specialised training at a tertiary centre and two months at a secondary centre. In 2020, the programme was extended to 36 months, with nine months of comprehensive training at a tertiary centre, 12 months of training in a secondary centre, and 15 months of subspecialty training. This expansion led to a demand for ongoing corneal surgical training during the year-long secondary centre placement, to better prepare fellows for the final phase of their training.

Although distance education programmes were available in the form of classes and seminars, they fell short of providing live surgical training. To address this challenge, we developed an innovative and cost-effective model for remote wet laboratory surgical training focused on penetrating keratoplasty (PK) suturing techniques. This model aimed to equip fellows with the skills and expertise necessary to excel in corneal surgery.

## Remote wet lab training model

The remote wet lab model to train fellows in penetrating keratoplasty suturing consists of three components:

- Theoretical classes/ didactic lectures
- Remote demonstration from the tertiary centre wet lab
- Setting up the wet lab at the secondary centre

Essential items for establishing a wet lab at secondary and tertiary centres include:

1. Artificial anterior chambers
2. Research-grade corneas
3. Suturing material (10-0 nylon)
4. Suturing instruments:
  - Hoskins forceps
  - Straight suture-tying forceps
  - Curved suture-tying forceps
  - Needle-holding forceps
  - Vannas scissors
5. External high-definition camera connected to a desktop
6. Video conferencing app (e.g., Zoom)

**Figure 1:**
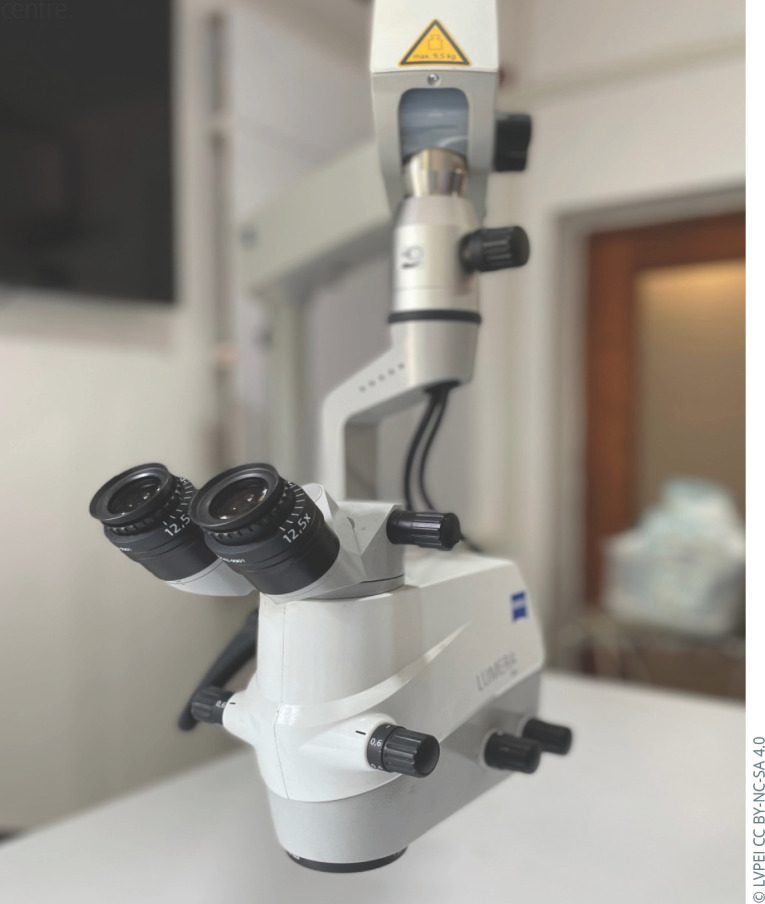
Operating microscope with camera set up at tertiary centre.

Additionally, at the tertiary centre, an operating microscope with a camera (refer to Figure 1) is required. At the secondary centre, a smartphone adaptor for the side scope of the operating microscope is needed.

### Pre-wet lab lecture

The first step was a detailed lecture for fellows covering the important concepts of penetrating keratoplasty suturing, including the technique and challenges. We also showed surgical video clips of penetrating keratoplasty performed on patients. The Video conferencing used Zoom, and was followed by a question-and-answer session enabling fellows to raise any concerns about surgical techniques.

### Remote demonstration from the tertiary centre wet lab

After the introductory lecture, penetrating keratoplasty suturing on a research-grade cornea mounted on a Baron artificial anterior chamber model number K20-2125 in the tertiary centre TC wet lab was demonstrated live by the trainer. For this set-up, we had two cameras installed – one from the microscope to provide the surgeon's view and another from the external camera on the desk. This way, fellows could observe the hand positioning and instrumentation, as well as the suturing technique, on their screens.

### Wet lab set-up at the secondary centre

A similar dual-feed relay system was planned for the secondary centre. However, due to limitations in obtaining a direct video relay from the microscope at the secondary centre, we developed an innovative, cost-effective smartphone adaptor device called Focus.

An external view from the laptop's HD camera was positioned at a one-meter distance from the operating table, providing a clear perspective of the hand positioning of fellows and the way they handled instruments.

Before the session, research-grade corneas, artificial anterior chamber, focus devices and instructions for assembly were sent to the secondary centres. During the session, each fellow performed a full penetrating keratoplasty suturing. All their actions were displayed on two screens for faculty to observe and provide feedback. Regular real-time feedback on suturing techniques was given, allowing for necessary adjustments and appropriate modifications.

**Figure 2:**
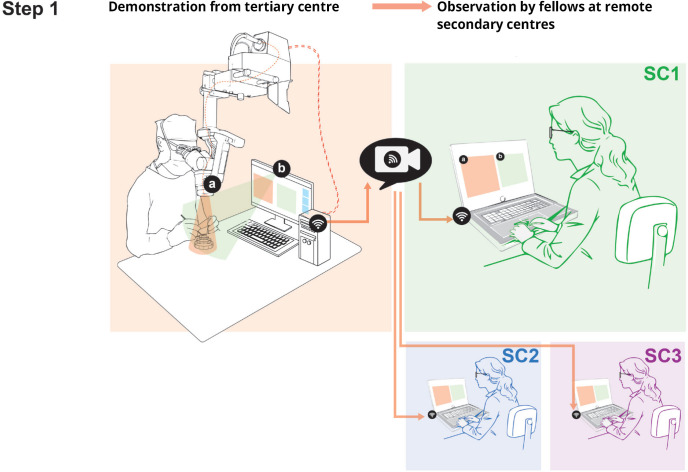
Remote wet lab setup at the tertiary centre.

In this remote wet lab set-up, a trainer demonstrates penetrating keratoplasty suturing on the research-grade cornea. Figure 2 illustrates the workflow setup. The visual is transmitted through two sources : (a) shows the camera feed from the operating microscope and (b) shows the external camera from the desktop computer. Both the screens are relayed to the fellows at three secondary centres (SC1, SC2, and SC3) via Zoom, allowing each fellow to see both views on their screens.

**Figure 3:**
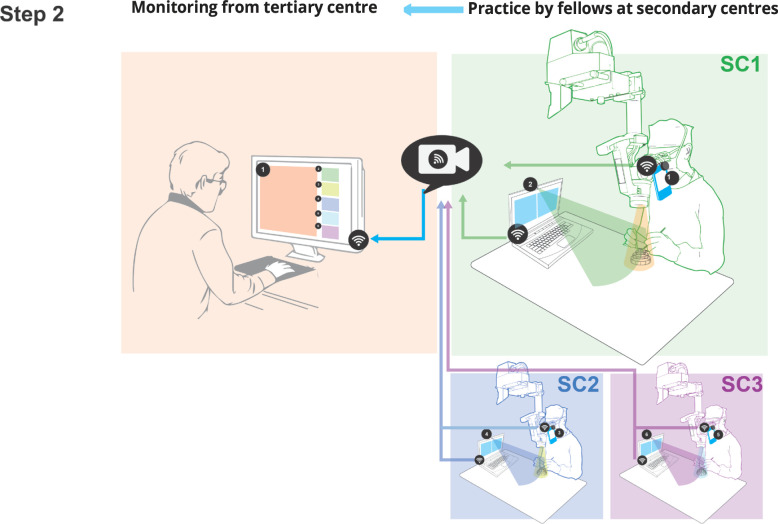
Remote wet lab setup at the secondary centres.

Figure 3 illustrates the feed arrangement at the secondary centres. The feed from the trainees' side scope, connected with a smartphone (with Focus) and the display from a laptop HD camera, are relayed to the trainer monitor at the tertiary centre. Each trainee's two screens, showing the surgeon's view and the external view, are relayed to the trainer. This setup enables trainers to provide real-time feedback to fellows, so they can improve their suturing techniques and make the necessary adjustments.

## Key learnings and way forward

Telementoring and virtual wet lab sessions emerged as vital tools for continuous medical education during the pandemic. While reliance on virtual platforms has decreased post-pandemic, they remain essential in our educational model, ensuring fellows' ongoing engagement in corneal procedures during their second year of study and enhancing their training beyond cataract surgeries.^5,6^ Besides penetrating keratoplasty, there are training modules for ocular surface procedures, corneal tear repair, perforation management, and donor tissue preparation for endothelial keratoplasty. The cost-efficient nature of our approach, with a manufacturing expense of approximately US $3 for the Focus device, permits easy replication for remote education, saving time and costs by eliminating the need for fellows to travel from remote secondary centres.

There remain occasional connectivity issues due to low rural internet speeds. In some secondary centres, dual video relay is not possible because of the lack of side scopes in operating microscopes.

It would be useful to include faculty-graded objective assessment alongside fellows' subjective feedback. While replicating an in-person instructor's intuitive feedback is a challenge, the model's effectiveness remains evident.

In conclusion, this low-cost remote wet lab model has proved to be feasible and effective for corneal surgical training. Regular and more frequent sessions hold promise for a more significant future impact.

**About the Focus device**

**Figure:**
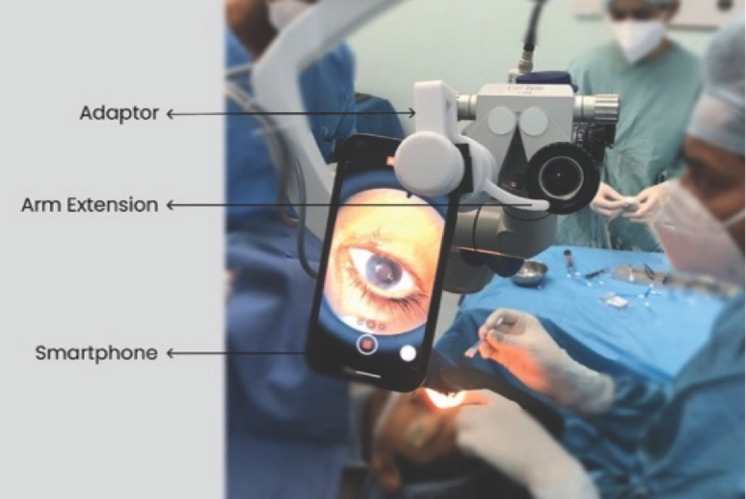

The Focus device was developed in-house at the Centre for Technology and Innovation, LV Prasad Eye Institute at a cost of about Rs 250 (roughly US $3). The device can be attached to the assistant scope, providing the surgeon's view.

The device has two main parts: the adaptor and the screw. It was created using Solidworks 3D design software and prepared for 3D printing using Cura software. The design was translated into G-code machine language, saved on an SD card, and put into the Ultimaker 2+ 3D printer.

The device is made from a type of plastic called PLA (polylactic acid). After it is 3D printed, extra portions, or supports, are removed using pliers and cutters. To ensure the smartphone does not slip from the microscope eye piece, a 4 mm thick, soft foam padding is added. An arm extension is also provided as additional support.

Once the focus device is attached to the side scope of the operating microscope, doctors can use their smartphones to live-stream operations in real-time.
